# Supplementary material for: Iterative improvement in the automatic modular design of robot swarms
Source: PeerJ Comput Sci. 2020 Dec 7;6:e322. doi: 10.7717/peerj-cs.322 (PMC7924708; doi:10.7717/peerj-cs.322)
Supplement: Supplemental Information 3 [file peerj-cs-06-322-s003.zip › argos3/doc/api/standalone/a00524_source.html]

ARGoS: plugins/robots/generic/simulator/differential\_steering\_default\_sensor.cpp Source File


- Main Page
- Related Pages
- Namespaces
- Classes
- Files

- File List
- File Members

# plugins/robots/generic/simulator/differential\_steering\_default\_sensor.cpp

Go to the documentation of this file.

```
00001 
00007 #include <argos3/core/simulator/simulator.h>
00008 #include <argos3/plugins/simulator/entities/wheeled_entity.h>
00009 #include <argos3/core/simulator/entity/composable_entity.h>
00010 
00011 #include "differential_steering_default_sensor.h"
00012 
00013 namespace argos {
00014 
00015    /****************************************/
00016    /****************************************/
00017 
00018    CDifferentialSteeringDefaultSensor::CDifferentialSteeringDefaultSensor() :
00019       m_pcWheeledEntity(NULL),
00020       m_pcRNG(NULL),
00021       m_bAddNoise(false) {}
00022 
00023    /****************************************/
00024    /****************************************/
00025 
00026    void CDifferentialSteeringDefaultSensor::SetRobot(CComposableEntity& c_entity) {
00027       try {
00028          m_pcWheeledEntity = &(c_entity.GetComponent<CWheeledEntity>("wheels"));
00029          if(m_pcWheeledEntity->GetNumWheels() != 2) {
00030             THROW_ARGOSEXCEPTION("The differential steering sensor can be associated only to a robot with 2 wheels");
00031          }
00032          m_pfWheelVelocities = m_pcWheeledEntity->GetWheelVelocities();
00033          m_sReading.WheelAxisLength = 100.0f * Distance(m_pcWheeledEntity->GetWheelPosition(0),
00034                                                         m_pcWheeledEntity->GetWheelPosition(1));
00035          m_pcWheeledEntity->Enable();
00036       }
00037       catch(CARGoSException& ex) {
00038          THROW_ARGOSEXCEPTION_NESTED("Error setting differential steering sensor to entity \"" << c_entity.GetId() << "\"", ex);
00039       }
00040    }
00041 
00042    /****************************************/
00043    /****************************************/
00044 
00045    void CDifferentialSteeringDefaultSensor::Init(TConfigurationNode& t_tree) {
00046       try {
00047          CCI_DifferentialSteeringSensor::Init(t_tree);
00048          /* Parse noise range */
00049          GetNodeAttributeOrDefault(t_tree, "vel_noise_range",  m_cVelNoiseRange,  m_cVelNoiseRange);
00050          GetNodeAttributeOrDefault(t_tree, "dist_noise_range", m_cDistNoiseRange, m_cDistNoiseRange);
00051          if(m_cVelNoiseRange.GetSpan() != 0 ||
00052             m_cDistNoiseRange.GetSpan() != 0) {
00053             m_bAddNoise = true;
00054             m_pcRNG = CRandom::CreateRNG("argos");
00055          }
00056       }
00057       catch(CARGoSException& ex) {
00058          THROW_ARGOSEXCEPTION_NESTED("Initialization error in default differential steering sensor", ex);
00059       }
00060    }
00061 
00062    /****************************************/
00063    /****************************************/
00064    
00065    void CDifferentialSteeringDefaultSensor::Update() {
00066       m_sReading.VelocityLeftWheel = m_pfWheelVelocities[0] * 100.0f;
00067       m_sReading.VelocityRightWheel = m_pfWheelVelocities[1] * 100.0f;
00068       m_sReading.CoveredDistanceLeftWheel = m_sReading.VelocityLeftWheel * CPhysicsEngine::GetSimulationClockTick();
00069       m_sReading.CoveredDistanceRightWheel = m_sReading.VelocityRightWheel * CPhysicsEngine::GetSimulationClockTick();
00070       if(m_bAddNoise) {
00071          m_sReading.VelocityLeftWheel  += m_pcRNG->Uniform(m_cVelNoiseRange);
00072          m_sReading.VelocityRightWheel += m_pcRNG->Uniform(m_cVelNoiseRange);
00073          m_sReading.CoveredDistanceLeftWheel  += m_pcRNG->Uniform(m_cDistNoiseRange);
00074          m_sReading.CoveredDistanceRightWheel += m_pcRNG->Uniform(m_cDistNoiseRange);
00075       }
00076    }
00077 
00078    /****************************************/
00079    /****************************************/
00080 
00081    void CDifferentialSteeringDefaultSensor::Reset() {
00082       m_sReading.VelocityLeftWheel         = 0.0f;
00083       m_sReading.VelocityRightWheel        = 0.0f;
00084       m_sReading.CoveredDistanceLeftWheel  = 0.0f;
00085       m_sReading.CoveredDistanceRightWheel = 0.0f;
00086    }
00087 
00088    /****************************************/
00089    /****************************************/
00090 
00091    REGISTER_SENSOR(CDifferentialSteeringDefaultSensor,
00092                    "differential_steering", "default",
00093                    "Carlo Pinciroli [ilpincy@gmail.com]",
00094                    "1.0",
00095                    "A generic differential steering sensor.",
00096                    "This sensor returns the current position and orientation of a robot. This sensor\n"
00097                    "can be used with any robot, since it accesses only the body component. In\n"
00098                    "controllers, you must include the ci_differential_steering_sensor.h header.\n\n"
00099                    "REQUIRED XML CONFIGURATION\n\n"
00100                    "  <controllers>\n"
00101                    "    ...\n"
00102                    "    <my_controller ...>\n"
00103                    "      ...\n"
00104                    "      <sensors>\n"
00105                    "        ...\n"
00106                    "        <differential_steering implementation=\"default\" />\n"
00107                    "        ...\n"
00108                    "      </sensors>\n"
00109                    "      ...\n"
00110                    "    </my_controller>\n"
00111                    "    ...\n"
00112                    "  </controllers>\n\n"
00113                    "OPTIONAL XML CONFIGURATION\n\n"
00114                    "It is possible to add uniform noise to the sensor, thus matching the\n"
00115                    "characteristics of a real robot better. You can add noise through the\n"
00116                    "attributes 'vel_noise_range' and 'dist_noise_range'.\n"
00117                    "Attribute 'vel_noise_range' regulates the noise range on the velocity returned\n"
00118                    "by the sensor. Attribute 'dist_noise_range' sets the noise range on the\n"
00119                    "distance covered by the wheels.\n"
00120                    "value for rotation.\n\n"
00121                    "  <controllers>\n"
00122                    "    ...\n"
00123                    "    <my_controller ...>\n"
00124                    "      ...\n"
00125                    "      <sensors>\n"
00126                    "        ...\n"
00127                    "        <differential_steering implementation=\"default\"\n"
00128                    "                               vel_noise_range=\"-0.1:0.2\"\n"
00129                    "                               dist_noise_range=\"-10.5:13.7\" />\n"
00130                    "        ...\n"
00131                    "      </sensors>\n"
00132                    "      ...\n"
00133                    "    </my_controller>\n"
00134                    "    ...\n"
00135                    "  </controllers>\n\n"
00136                    "OPTIONAL XML CONFIGURATION\n\n"
00137                    "None.\n",
00138                    "Usable"
00139                   );
00140 
00141 }
```

---

Generated on 10 Jul 2018 for ARGoS by 
 1.6.1
